# HIPPO signaling-related signature for predicting prognosis and therapeutic response in gastric cancer

**DOI:** 10.3389/fphar.2022.1096055

**Published:** 2023-01-11

**Authors:** Rui Jiang, Jinghua Wang, Jun Liang, Daihua Lin, Qiuxian Mao, Siyi Cheng, Shengjun Huang, Shuangshuang Tong, Yanlin lyu, Rui Wei, Qizhou Lian, Hao Chen

**Affiliations:** ^1^ Department of Gastroenterology, Guangdong Provincial People’s Hospital, Guangdong Academy of Medical Sciences, Guangzhou, China; ^2^ Department of Hematology, Guangdong Provincial People’s Hospital, Guangdong Academy of Medical Sciences, Guangzhou, China; ^3^ Department of Critical Care Medicine, Guangdong Provincial Geriatrics Institute, Guangdong Provincial People’s Hospital, Guangdong Academy of Medical Sciences, Guangzhou, China; ^4^ Prenatal Diagnostic Center, Guangdong Second Provincial General Hospital, Guangzhou, China; ^5^ School of Bioscience and Bioengineering, South China University of Technology, Guangzhou, China; ^6^ School of Medicine, South China University of Technologyy, Guangzhou, China; ^7^ Shantou University Medical College, Shantou, China; ^8^ Department of Medicine, Li Ka Shing Faculty of Medicine, The University of Hong Kong, Hong Kong SAR, China; ^9^ Faculty of Synthetic Biology, Shenzhen Institute of Advanced Technology, Chinese Academy of Sciences, Shenzhen, China; ^10^ Cord Blood Bank, Guangzhou Institute of Eugenics and Perinatology, Guangzhou Women and Children’s Medical Center, Guangzhou Medical University, Guangzhou, China; ^11^ State Key Laboratory of Pharmaceutical Biotechnology, The University of Hong Kong, Hong Kong SAR, China

**Keywords:** gastric cancer, hippo signaling pathway, prognostic prediction, drug sensitivity analysis, therapeutic response

## Abstract

**Background:** Gastric cancer (GC) is a multifactorial progressive disease with high mortality and heterogeneous prognosis. Effective prognostic biomarkers for GC were critically needed. Hippo signaling pathway is one of the critical mechanisms regulating the occurrence and development of GC, and has potential clinical application value for the prognosis and treatment of GC patients. However, there is no effective signature based on Hippo signaling pathway-related genes (HSPRGs) to predict the prognosis and treatment response of GC patients. Our study aimed to build a HSPRGs signature and explore its performance in improving prognostic assessment and drug therapeutic response in GC.

**Methods:** Based on gene expression profiles obtained from The Cancer Genome Atlas (TCGA) database, we identified differentially expressed HSPRGs and conducted univariate and the least absolute shrinkage and selection operator (LASSO) Cox regression analysis to construct a multigene risk signature. Subsequently, the Kaplan-Meier curve and receiver operating characteristic (ROC) were performed to evaluate the predictive value of the risk signature in both training and validation cohort. Furthermore, we carried out univariate and multivariate Cox regression analysis to investigate the independent prognostic factors and establish a predictive nomogram. The enriched signaling pathways in risk signature were analyzed by gene set enrichment analysis (GSEA). Tumor immune dysfunction and exclusion (TIDE) and drug sensitivity analysis were performed to depict therapeutic response in GC.

**Results:** In total, 38 differentially expressed HSPRGs were identified, and final four genes (*DLG3, TGFB3, TGFBR1, FZD6*) were incorporated to build the signature. The ROC curve with average 1-, 3-, and 5-year areas under the curve (AUC) equal to .609, .634, and .639. Clinical ROC curve revealed that risk signature was superior to other clinicopathological factors in predicting prognosis. Calibration curves and C-index (.655) of nomogram showed excellent consistency. Besides, in the immunotherapy analysis, exclusion (*p* < 2.22 × 10^–16^) and microsatellite instability (*p* = .0058) performed significantly differences. Finally, our results suggested that patients in the high-risk group were more sensitive to specific chemotherapeutic agents.

**Conclusion:** Results support the hypothesis that Hippo-related signature is a novel prognostic biomarker and predictor, which could help optimize GC prognostic stratification and inform clinical medication decisions.

## 1 Introduction

Gastric cancer (GC) is an aggressive gastrointestinal malignancy, ranking fourth in cancer-related death worldwide (Thrift and El-Serag, 2020), and seriously threatens human health. Radical resection of GC is currently the main treatment method for GC (Caruso et al., 2016), but the curative effect of surgery for advanced GC is not high (Smyth et al., 2020). As the field of GC treatment has made great strides, the morbidity and mortality rates have progressively decreased in recent years (Wong et al., 2021). However, the mortality rate remains high due to the late presentation of GC and the prognosis of GC patients is still relatively poor. At present, molecular targeted therapy has been one of the most promising treatments of various cancers (Jahangir and Polin, 2016). Due to the lack of a complete understanding of the molecular mechanism, the effective targeted therapy for the clinical treatment of GC is less than that of other cancers (Grech et al., 2015). Therefore, understanding the biological pathways leading to the development of GC and developing new prognostic stratification of GC patients based on this will be crucial for improving GC prognosis and formulating appropriate treatment strategies.

As a critical tumor suppressor pathway, Hippo tumor suppressor pathway plays a vital role in regulating cell proliferation, tissue damage and regeneration, tumorigenesis, development, metastasis and therapy (Gu et al., 2021). Previous studies have shown that hippo pathway effectors Yes-associated protein (YAP) and transcriptional co-activator with PDZ-binding motif (TAZ) play a particularly important role in GC and are closely related to prognosis (Seeneevassen et al., 2022). In addition, Helicobacter pylori can also activate proliferation genes and inflammatory cytokines by inducing YAP, a key effector of Hippo pathway (Wu et al., 2019). Hippo signaling pathway-Related Genes (HSPRGs) might promote the growth and metastasis of gastric cancer by inhibiting Hippo pathway signaling to support YAP, which still requires further confirmation. Traditional surgical treatment for gastric cancer has low curative power. Based on the further study of the molecular mechanism of gastric cancer, more chemotherapy drugs and molecular targeted drugs are emerging. For example, verteporfin, Sitagliptin and amphiregulin (AREG) are several promising anti-GC drugs, targeting to inhibit the activation of the key Hippo pathway effector YAP (Qiao et al., 2018; Yong et al., 2021). The identification of drug sensitivity for gastric cancer patients still needs continuous exploration for clinical exploration and drug guidance. Recent researches have also proven that the activity of the Hippo pathway is closely related to various anti-tumor immune responses (Moroishi et al., 2016), which suggest new and innovative strategies for the development of immunotherapy.

Given the critical role of the Hippo signaling pathway in GC growth control and inhibition (Kang et al., 2016), we systematically analyzed the differentially expressed HSPRGs between GC patients and health based on TCGA cohort, and then constructed a prognostic signature related to the Hippo pathway. The external dataset GEO (GSE84433) was used as a validation cohort to illustrate its prognostic efficacy. Further, we explored the predictive effect of HSPRGs-related signature on response to immunotherapy and chemotherapy. We hypothesis that the Hippo-related signature has a certain impact on the prognosis of GC, and can be used for GC prognostic evaluation and medication guidance.

## 2 Materials and methods

### 2.1 Data collection

Complete RNA-seq transcriptome and clinical data sets of GC patients were downloaded from The Cancer Genome Atlas (TCGA, https://portal.gdc.cancer.gov/) and the Gene Expression Omnibus (GEO, https://www.ncbi.nlm.nih.gov/geo/) databases. In total, 407 patients from The Cancer Genome Atlas Stomach Adenocarcinoma (TCGA-STAD) were chose as training set, 357 patients from GSE84433 (Yoon et al., 2020) were selected for validation. All gene expression datasets and clinical data sets of GC are publicly available. Totally 108 HSPRGs were obtained from the Kyoto Encyclopedia of Genes and Genomes (KEGG, https://www.kegg.jp/kegg/), a website can understand high-level functions and utilities of the Hippo signaling pathway genes. However, 19 genes related to Hippo signaling pathway lacked the gene expression data in TCGA dataset. As a result, 89 hippo-related genes with expression data were chosen for further analysis.

### 2.2 Differential expression and protein-protein-interaction analysis

We extracted the transcriptome profiling and clinical data of HSPRGs from TCGA dataset. Subsequently, the differential expression HSPRGs in the TCGA dataset were selected using the “limma” package, under the filter of |log fold change (logFC)| ≥ .5 and false discovery rate (FDR) < .05. The up-regulated, down-regulated and indifferently expressed genes of the Hippo pathway were represented by volcano plots. Differentially expressed HSPRGs in normal and GC tissues were visualized into heatmap plots. Additionally, protein-protein-interaction (PPI) analysis was derived from the STRING database (http://www.string-db.org/) to visualize protein-protein interactions related to differentially expressed HSPRGs. Disconnected nodes are hidden and a minimum interaction score of .700 was required in drawing PPI.

### 2.3 Construction and validation of the HSPRGs-Based prognostic Signature

To figure out significantly prognostic HSPRGs associated with OS, we conducted a univariate Cox regression analysis in TCGA dataset using the “survival” package. Then, we performed the least absolute shrinkage and selection operator (LASSO) to select reliable predictors and applied multivariate Cox regression analysis to construct a multigene prognostic risk signature and calculate the risk score corresponding to each sample, using the “glmnet” package. The risk score for each patient was calculated as following formula: $risk\;score=\overset{n}{\underset{i=1}{\sum }}coef_{i}\;*\;x_{i}$ , with coef_i_ representing the regression coefficient, and x_i_ representing the expression level of each gene. Patients with GC in training set were stratified into high- and low-risk groups base on the median risk score. The plots of risk score distribution and survival status were explored in each GC patient in the high- and low-risk groups. Kaplan-Meier survival curve and log-rank test were applied to assess survival differences between the two groups, using the “survival” and “survminer” package. To further appraise the prognostic accuracy of the signature, we constructed receiver operating characteristic (ROC) curves for 1-, 3-, and 5-year survival and calculated the area under the curve (AUC) values. Besides, the same statistical operations were implemented in the GSE84433 validation set, including calculation of risk scores, subsequent group division, and validation of model stability.

### 2.4 Independence prognostic analysis of risk signature

To identify the clinical application and evaluate the ability for independent prognostic analyses of risk prognostic signature, we performed univariate and multivariate Cox regression analyses with risk score and other clinical factors (including age, gender, grade and stage), which was visualized with a heatmap (* represents *p* < .05, ** represents *p* < .01, *** represents *p* < .001). Moreover, we carried out a ROC curve to compare the prognostic effects between risk score and other clinical factors.

### 2.5 Predictive nomogram construction

To visually describes the impact of prognostic factors and predicted 1-, 3- and 5-year survival, age, stage and risk score which were significant difference in the above analysis of clinical independence were included to construct a robust nomogram, using the “rms” package. Furthermore, calibration curves and C-index were used to assess the accuracy and performance of the nomogram.

### 2.6 Gene set enrichment analysis (GSEA) of signature

GSEA was conducted to uncover the Kyoto Encyclopedia of Genes and Genomes (KEGG) differentially enriched signaling pathways of the HSPRGs between the low- and high-risk groups, using “clusterprofiler” package. The gene set was obtained from the “c2.cp.kegg.v7.4.symbols.gmt” file in GSEA software (Mootha et al., 2003) (http://software.broadinstitute.org/gsea/). Only the first five more prominent enriched pathways in high- and low-risk groups were shown respectively.

### 2.7 Tumor immune dysfunction and exclusion (TIDE)

TIDE is a computational approach based on modeling tumor immune evasion mechanisms to predict responsiveness to immunotherapy (Jiang et al., 2018). We evaluated the impact of expression of the risk signature in the immune therapy response, including microsatellite instability (MSI), exclusion and TIDE, and visualized by violin diagram (* represents *p* < .05, ** represents *p* < .01, *** represents *p* < .001). TIDE scores related GC patients were sourced from TIDE analysis tool (http://tide.dfci.harvard.edu/).

### 2.8 Drug sensitivity analysis

Since not all patients with advanced GC are sensitive to chemotherapy, we investigated the chemotherapy response in different risk groups in GC. Predicting chemotherapeutic response for each sample by the half maximal inhibitory concentration (IC50) using “pRRophetic” package (Geeleher et al., 2014), based on Genomics of Drug Sensitivity in Cancer (GDSC) (https://www.cancerrxgene.org), currently the largest public pharmacogenomics database.

### 2.9 Statistical analysis

R software (version 4.1.1; https://www.r-project.org/) was used for all statistical analyses. For quantitative data, statistical significance was estimated using Student’s t-tests. Survival curve and ROC curve analyses were performed to examine the predictive accuracy of risk score, and the “pRRophetic” R package was implemented for chemotherapy response prediction. During all the result statistics, *p* < .05 was considered as statistically significant difference.

## 3 Results

### 3.1 Extraction and differential expression analysis of HSPRGs

**FIGURE 1:**
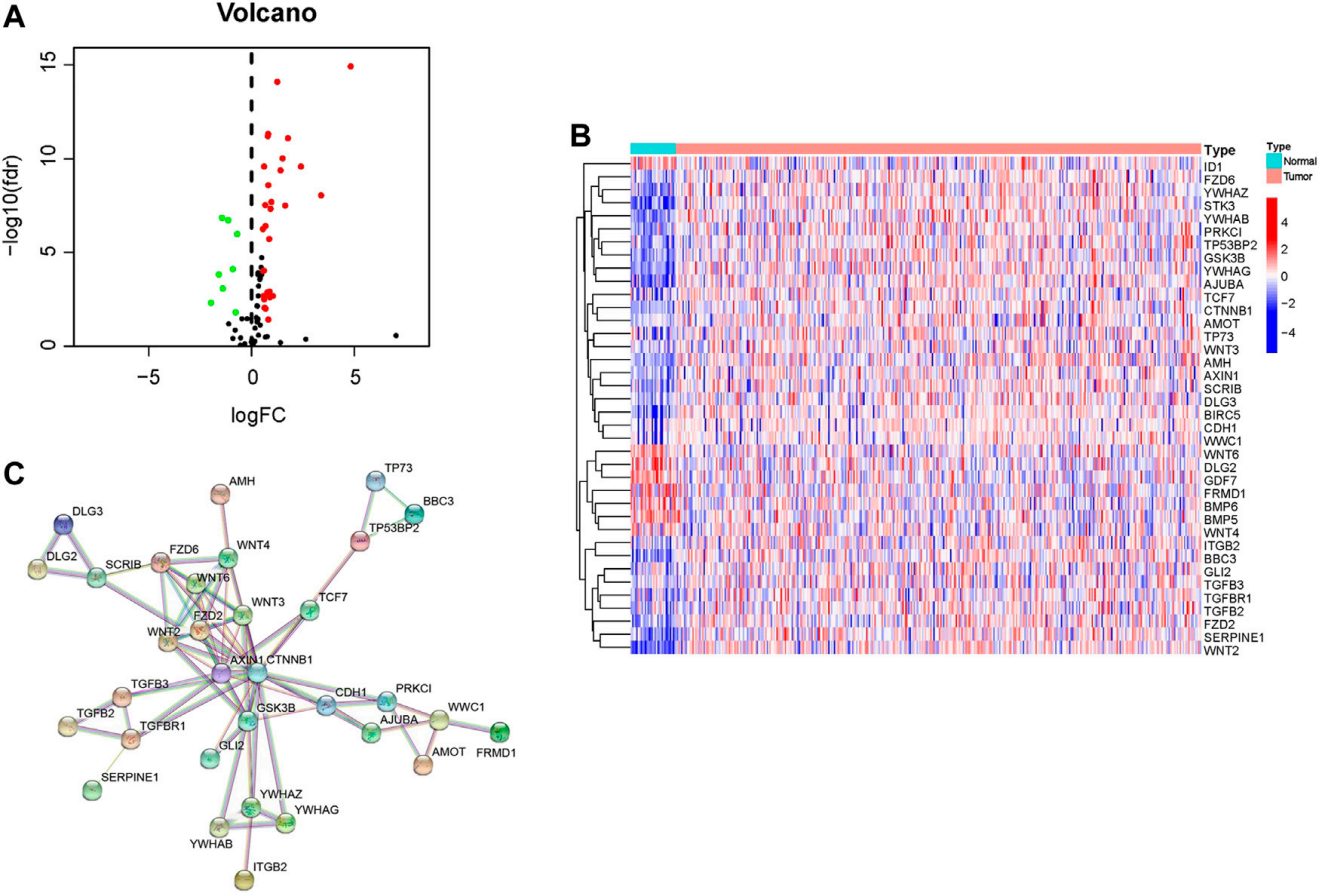
Identification of HSPRGs between GC and normal tissues. **(A)** Volcano plot depicting up-regulated differentially expressed HSPRGs in red, down-regulated differentially expressed HSPRGs in green, and non-significant genes in black. **(B)** Heatmap of differential expression HSPRGs. **(C)** The protein interaction biological relationship network of PPI.

Merging mRNA expression profiling of TCGA dataset and 108 HSPRGs derived from KEGG. 38 genes are significantly different expressed in GC. Volcano (red and green represented Up- and down-regulated different expression HSPRGs respectively, Figure 1A) and heatmap (Figure 1B) were drawn to show the differentially expressed genes in detail. In total, 38 connected nodes and 74 edges were shown in the PPI network diagram. Locating in the most central area of network, *CTNNB1, AXIN1, GSK3B, FZD2* and *WNT6* were identified as highly connected proteins. Connections of related functional proteins visualized in network might play an important role in the regulation of cells and their signaling (Figure 1C).

### 3.2 Establishment and validation of the prognostic signature

**FIGURE 2:**
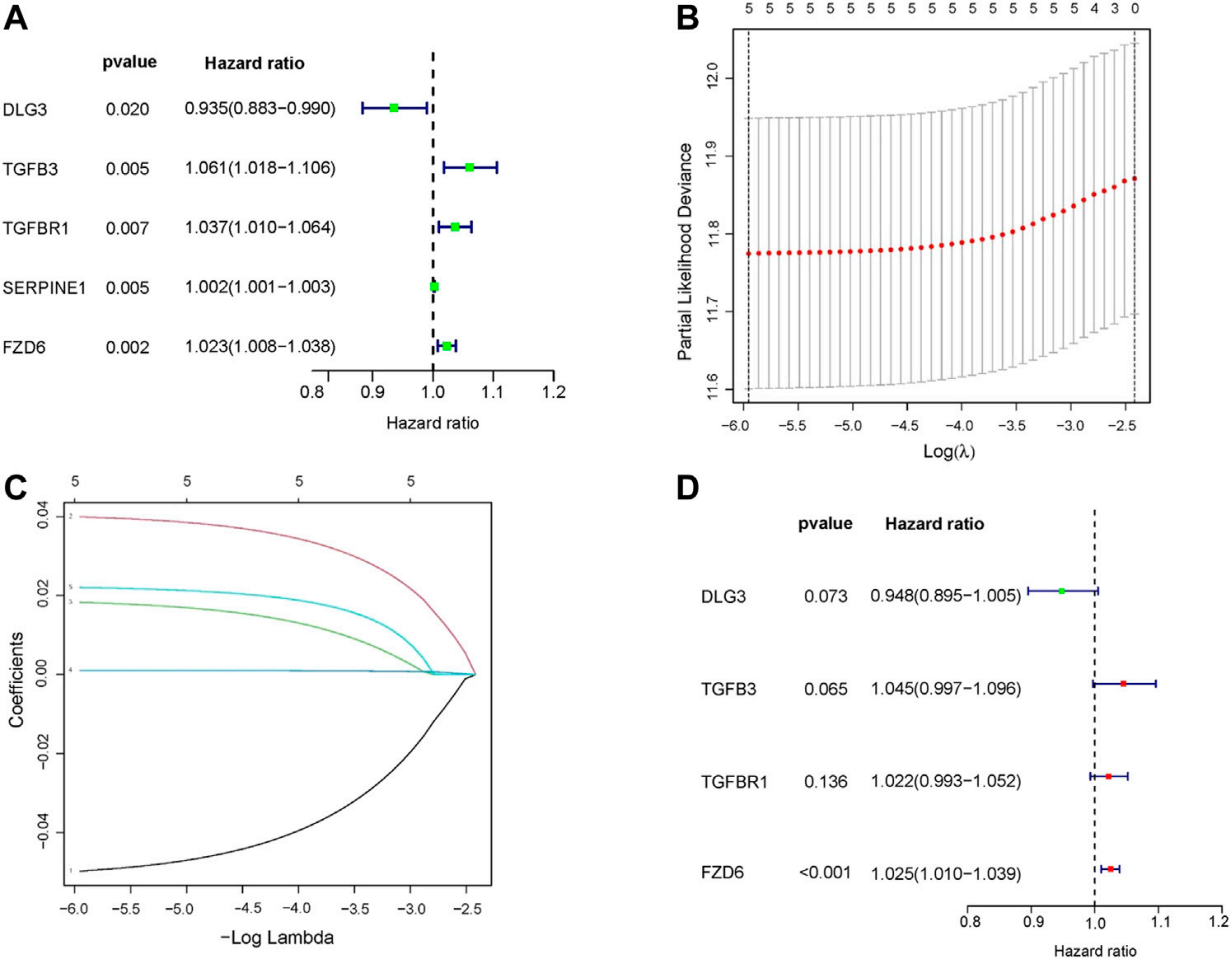
Construction of a HSPRGs signature for the prognosis of GC. **(A)** Univariate cox regression analysis **(B)** The minimum value was selected as the optimal parameter (λ) in the LASSO signature **(C)** LASSO coefficient map of prognosis-related HSPRGs. **(D)** Multivariate cox regression analysis.

**FIGURE 3:**
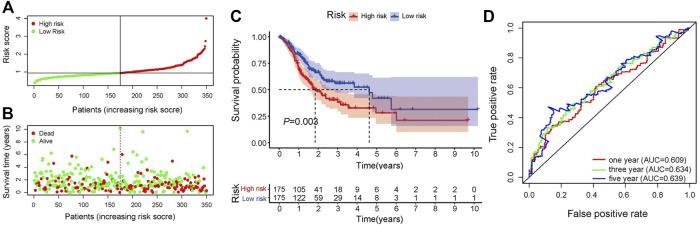
Validation of the signature. **(A)** Distribution of risk scores, patients are grouped by the median of risk scores. **(B)** Distribution of survival in high- and low-risk groups **(C)** Kaplan-Meier analysis of high- and low-risk groups. **(D)** 1-, 3-, and 5-year OS predictive ROC plots and AUC values.

**FIGURE 4:**
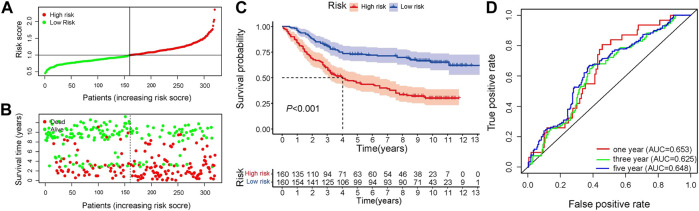
Validation of the independent dataset GSE84433. **(A)** Distribution of risk scores in GC patients **(B)** Distribution of survival status of high- and low-risk groups **(C)** Kaplan-Meier analysis of high- and low-risk group **(D)** 1-, 3-, and 5-year OS predictive ROC plots and AUC values.

In the training set, univariate Cox regression analysis (Figure 2A) indicated that five genes (*DLG3, TGFB3, TGFBR1, SERPINE1, FZD6*) were significantly linked to the OS in GC patients. Then, five prognostic genes mentioned above were filtered *via* the LASSO regression analysis (Figures 2B,C). Multivariate regression showed that only *FZD6* independently associated with GC prognosis (Figure 2D). Four genes (*DLG3, TGFB3, TGFBR1, FZD6*) selected by LASSO logistic regression to establish an excellent prognostic multigene signature. The risk score was calculated as follows: Risk score = (−.0531 × *DLG3* expression) + (.0444 × *TGFB3* expression) + (.0217 × *TGFBR1* expression) + (.0243 × *FZD6* expression). The median risk score calculated by the prognostic formula above was used to classify GC patients into high- and low-risk groups. The survival time of each patient showed that more deaths were found in the high-risk group (Figures 3A, B, 4A, 4B). Kaplan-Meier curves significantly indicated that worse OS in high-risk group patients compared to low-risk group in training (log-rank *p* = .003, Figure 3C) and validation sets (log-rank *p* < .001 for GSE84433 set, Figure 4C). Furthermore, in training and validation sets, the AUCs for 1-, 3-, and 5-year survival were .609, .634, .639 and .653, .625, .648, respectively (Figures 3D, 4D). In general, all results from the training and validation sets similarly revealed the excellent prognostic validity of our four genes signature.

### 3.3 Independent prognostic value of the HSPRGs signature

**FIGURE 5:**
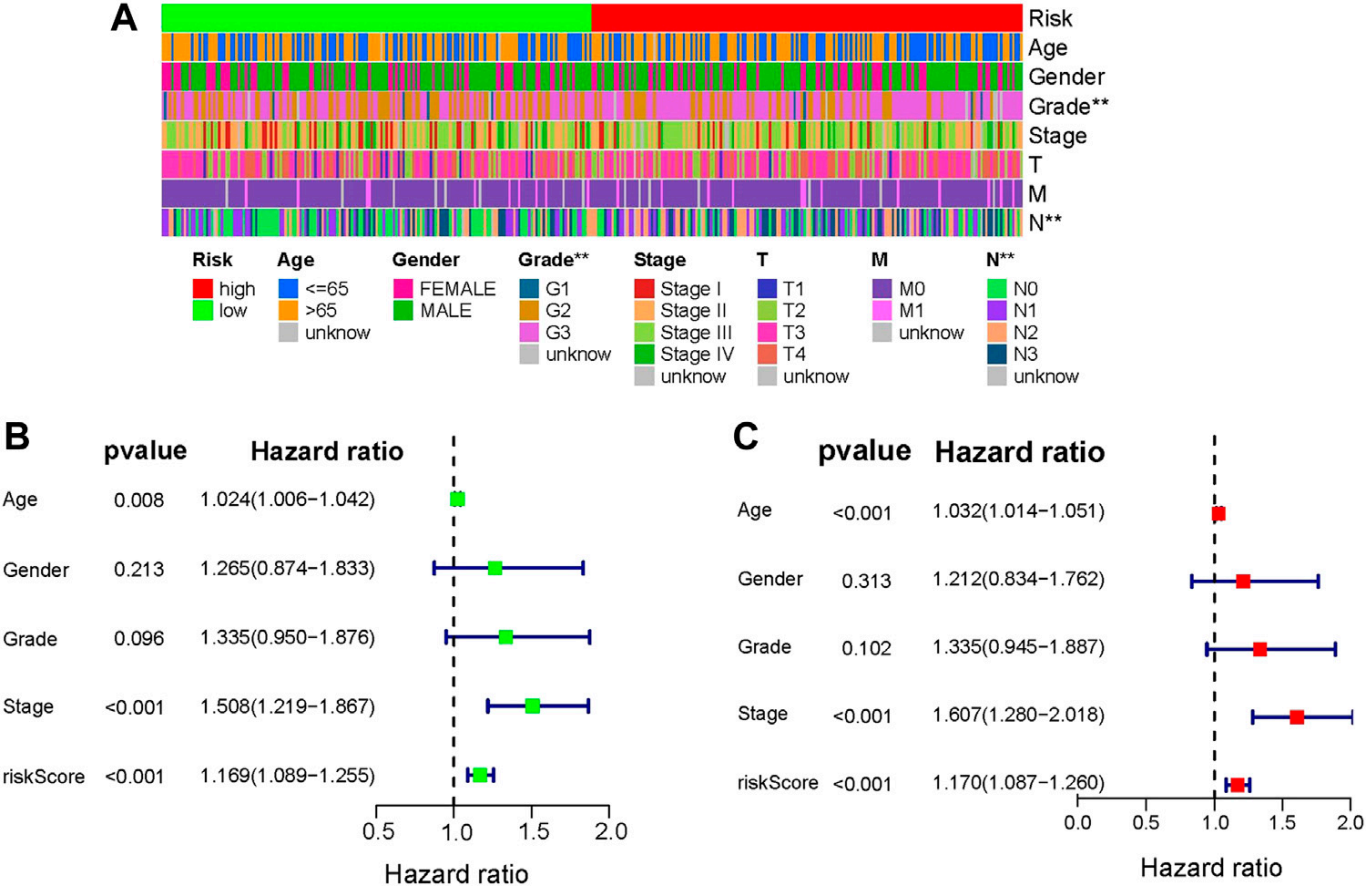
Analysis of clinical independence. **(A)** Heatmap of the relationship between risk score and clinical factors (* represents *p* <.05, ** represents *p* <.01, *** represents *p* <.001). **(B)** and **(C)** Univariate and multivariate cox analysis of risk score and other clinical factors.

**FIGURE 6:**
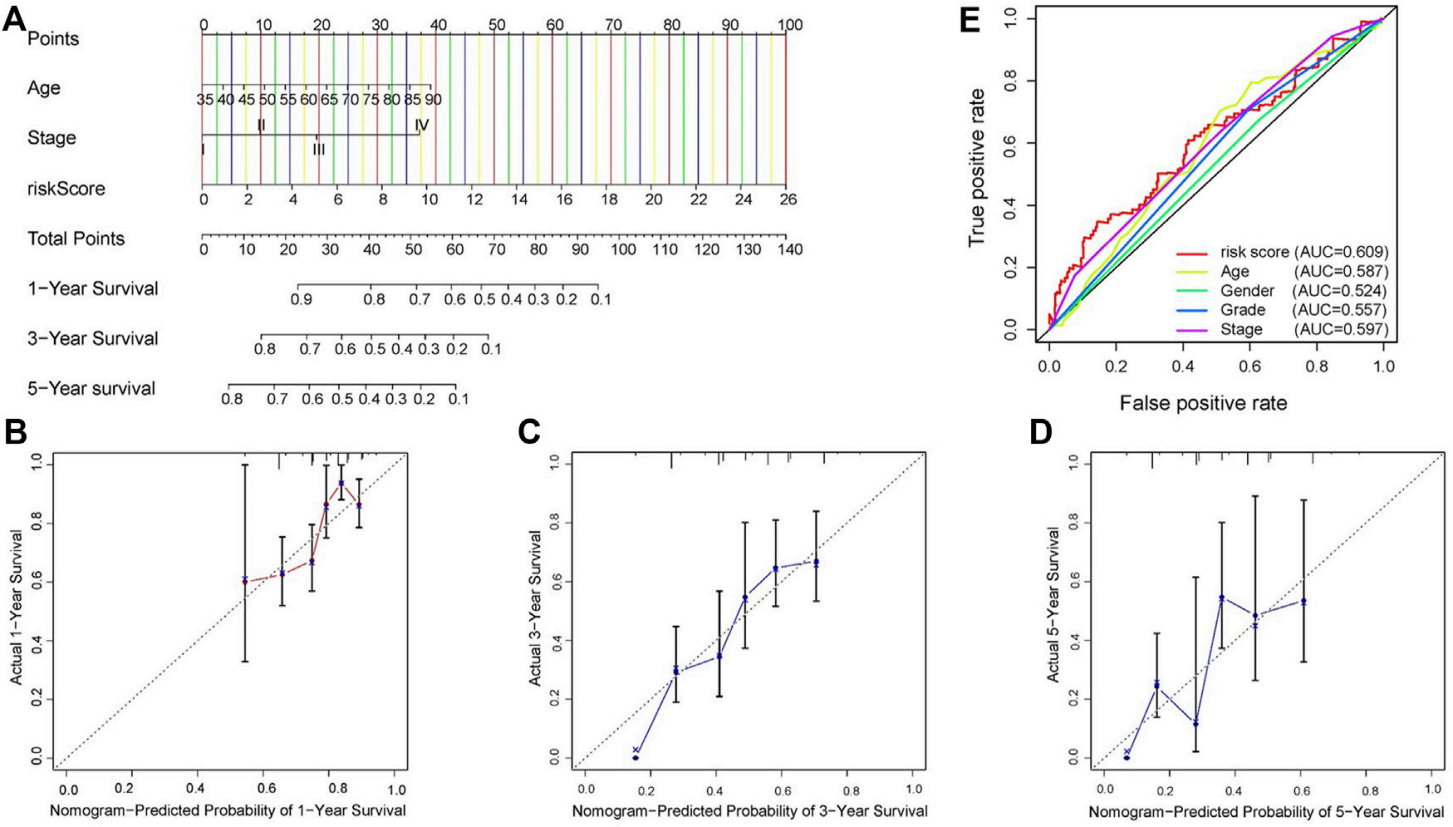
Construction of signature-based predictions. **(A)** Nomogram employed to predict 1-, 3-, and 5-year OS in GC patients. **(B–D)** Calibration curves representing the relationship between nomogram predicted survival probability and actual survival probability. **(E)** ROC curve and AUC values of signature and clinicopathological factors.

Following that, the independent prognostic role of risk signature was assessed using the analyses of univariate and multivariate Cox regression by comparing with other clinical factors including age, stage, grade, gender. Grade and N stage signed **(*p* < .01, Figure 5A) in heatmap, which indicated that Grade and N stage are significantly correlated with risk score. Univariate and multivariate analysis indicted that only age (HR = 1.024, *p* < .008; HR = 1.032, *p* < .001), stage (HR = 1.508, *p* < .001; HR = 1.607, *p* < .001) and risk score signature (HR = 1.169, *p* < .001; HR = 1.170, *p* < .001) were independent prognostic risk factors (Figures 5B, C). In the clinical independent ROC (Figure 6E), the AUC values for the risk score (AUC = .609) were higher than age (AUC = .587), stage (AUC = .597), grade (AUC = .557) and gender (AUC = .524). Thus, the four-gene signature we built was superior to other clinical variables in predicting OS of GC.

### 3.4 Construction and validation of predictive nomogram

In TCGA dataset, independent risk factors (Risk score, Age and T stage) were included in constructing a nomogram for effective prediction of survival in GC, based on the four-gene signature (Figure 6A). Calibration curves for 1-, 3-, and 5-year OS in GC patients were relatively close to the reference line, indicating excellent agreement between nomogram predictions and observed survival probabilities (Figures 6B–D). Moreover, the C-index of .655 also estimated the outstanding prediction performance of our nomogram.

### 3.5 GSEA

**FIGURE 7:**
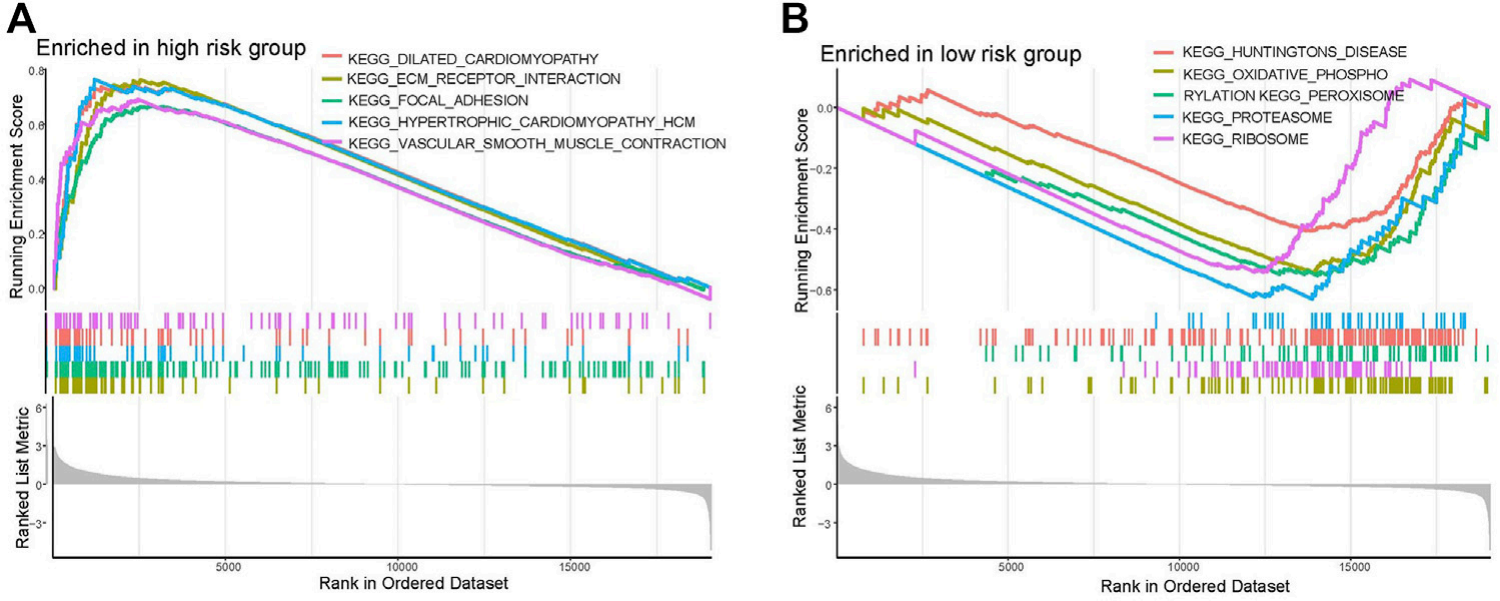
Gene set enrichment analysis in **(A)** high-risk and **(B)** low-risk groups.

The GSEA (c2.cp.kegg.v7.4.symbols.gmt) was applied for selecting the various pathways were active in high- and low-risk groups. High-risk group patients were mainly enriched in dilated cardiomyopathy, extracellular matrix–receptor (ECM-receptor) interaction, focal adhesions, hypertrophic cardiomyopathy (HCM) and vascular smooth muscle contraction pathways. Low-risk group patients were mainly enriched in huntingtons disease, oxidative phospho, peroxisome, proteasome and ribosome pathways (Figure 7).

### 3.6 Immunotherapy response in GC patients

**FIGURE 8:**
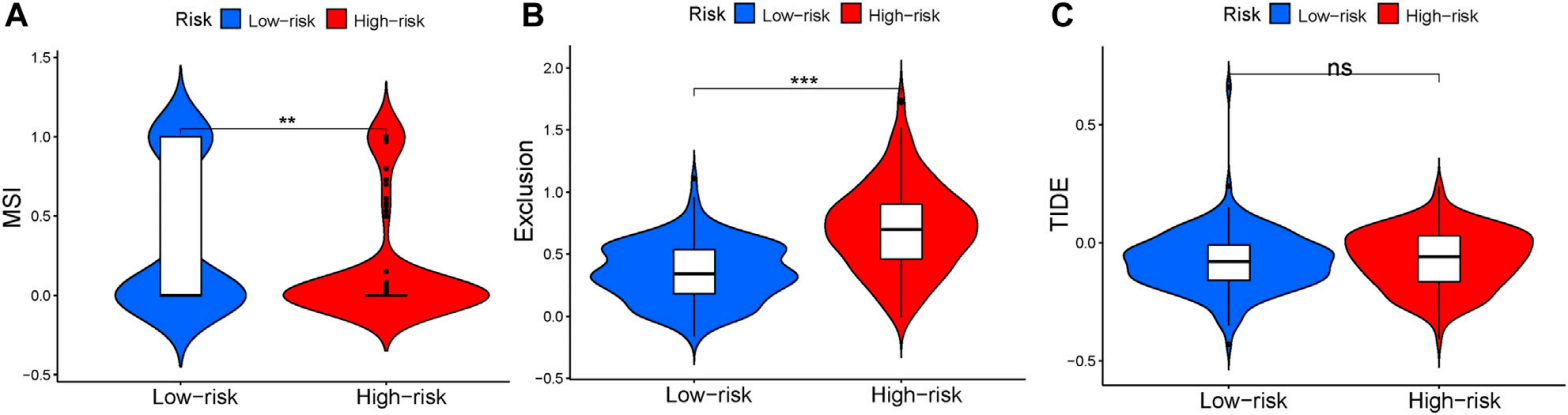
Tumor Immune Dysfunction and Exclusion between high- and low-risk groups. **(A)** Violin plot of MSI. **(B)** Violin plot of exclusion. **(C)** Violin plot of TIDE.

To predict the relationship between immunotherapy response and prognosis of risk scores using the TIDE algorithm. There was significant difference in microsatellite instability (MSI, *p* = .0058, Figure 8A) between high- and low-risk groups. The exclusion (*p* < 2.22 × 10^–16^,Figure 8B) was higher in high-risk group, manifesting that the low-risk group patients were more likely to be responsive to immunotherapy. Results concluded that the four-gene signature was potential for indicating the immunotherapy response in GC patients. However, there was no significant difference in TIDE (*p* = .27, Figure 8C).

### 3.7 Chemotherapeutic responses of high- and low-risk patients with GC

**FIGURE 9:**
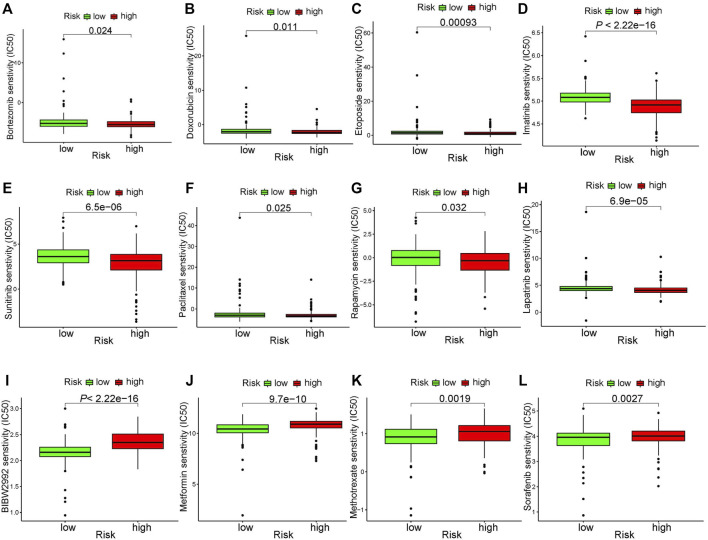
Chemotherapeutic drug sensitivity analysis. **(A–H)** drugs more sensitive in high-risk group. **(I–L)** drugs more sensitive in low-risk group.

In addition to immunotherapy, chemotherapy is currently the main adjuvant therapy for GC treatment. However, the gradual development of resistance to chemotherapy drugs in GC patients is a major problem. Therefore, it is vital important to select chemotherapeutic drugs for individual treatment of GC patients. Analysis of the sensitivity of chemotherapeutic drugs showed that patients in the high-risk group were more sensitive to Bortezomib, Doxorubicin, Etoposide, Imatinib, Lapatinib, Paclitaxel, Rapamycin, and Sunitinib (Figures 9A–H), while patients in the low-risk group were more sensitive to BIBW2992, Metformin, Methotrexate, and Sorafenib (Figures 9I–L). However, further experiments are required to verify these results.

## 4 Discussion

Overexpression of the Hippo signaling pathway effector YAP has been observed in GC, and research has discovered that Yap is a crucial factor for maintaining GC migration and viability (Yan et al., 2018; Kim et al., 2019). Past studies have shown the promise of HSPRGs as potential biomarkers in GC, but biomarkers for predicting prognosis and treatment response based on HSPRGs in GC have not been explored. Therefore, we are committed to research in this area, we found that our prognostic model can significantly distinguish high-risk GC patients with poor prognosis, which provides a novel reference to prognosis prediction of GC patients. Additionally, we collected the prognostic risk score and clinical factors of GC patients to present a new nomogram for evaluating the clinical prognosis of GC, which provides an important reference for clinical prognosis. At the same time, our model can examine the sensitivities of GC patients in different risk groups to immunotherapy and chemotherapy drugs, which has important application value for the clinical medication decisions of GC patients. As far as we know, we are pioneers in the construction of new models for prognostic prediction and treatment response based on genes associated with the Hippo pathway.

Prior researchers have established effective and predictive GC signatures related to immunity, ferroptosis and hypoxia genes. However, these prognostic models were limited by insufficient validation (Liu et al., 2020), limited to one sex of patients and a small number of samples for validation (Xu et al., 2021), and lack of drug response (Shao et al., 2021). The Hippo pathway has been shown to be involved in the progression of GC, but there is no study on the association between HSPRGs and GC prognosis. Our study established a novel signature, which may improve the predictive accuracy of the prognosis in GC patients and rationality in the selection of personal treatment strategies.

Genes constructing the risk signature are *DLG3, TGFB3, TGFBR1* and *FZD6*. The principal functions of these four genes and their association with cancer are as follows. According to previous studies, and the increase of Discs large homolog 3 (*DLG3*) gene can activate the Hippo signaling pathway (Chen et al., 2020a) and has the effect of suppressing further deterioration of GC (Li et al., 2020), oral squamous cell carcinoma, breast cancer (Liu et al., 2019), colon cancer and lung cancer. In addition to the Hippo pathway, *DLG3* can also inhibit the activation of the phosphatidylinositol 3-kinase/protein kinase B (PI3K/AKT) pathway (Liu et al., 2021), and the PI3K/AKT signaling pathway is implicated in the occurrence and progression of GC (Chen et al., 2020b). Interestingly, activation of the PI3K/AKT pathway can inhibit the Hippo pathway (Qian et al., 2021). However, whether DLG3 can affect GC through the PI3K/AKT pathway has not been explored. Transforming growth factor beta 3 (*TGFB3*) and transforming growth factor beta receptor 1 (TGFBR1) are both transforming growth factors, which are potent growth inhibitors that can effectively regulate cell growth, differentiation and apoptosis functions, and are frequently disturbed during the development of tumors, including GC (Chen et al., 2014). As target genes involved in the TGF-β pathways, *TGFB3* and *TGFBR1* may can accumulate YAP/TAZ proteins and inhibit Hippo pathway (Mohamed et al., 2019). *TGFB3* can regulate cell growth and differentiation, differentiation, migration (Lichtman et al., 2016) and the expression of *TGFB3* is related to the protection of gastric mucosa (Laverty et al., 2009), *TGFB3* exhibits abnormal colocalization and overexpression in human GC cells (Naef et al., 1997). Mutations in the *TGFBR1* gene induce tumorigenesis and promote tumor metastasis, which are associated with increased risk of breast, ovarian, and colorectal cancers (Lin et al., 2017). Frizzled receptor 6 (*FZD6*) is one of the key molecules of the Wingless-type MMTV integration site (Wnt) signaling network, repressing GC cell proliferation, mobility and invasion by activating Wnt pathway (Yan et al., 2016), *FZD6* receptor is involved in transduction of Wnt5A signaling in GC (Katoh, 2005) and Wnt ligands can Trigger YAP1 to affect the Hippo pathway (Kriz and Korinek, 2018). Notably, the expression of *FZD6* has a strong correlation with tumor malignancy prognosis. In addition, multivariate regression analysis of *FZD6* gene in our study was significant, which indicated that it was independently associated with prognosis, which means that it has the most potential to become a GC biomarker. These genes have various implications for cancer therapy, and the detailed mechanism of action of *DLG3, TGFB3, TGFBR1* and *FZD6* genes in GC has not been studied, and needs further researches to explore.

Immunotherapy has already been an effective treatment for GC (Li et al., 2021). TIDE algorithm was utilized to predict patient response to immunotherapy. MSI is more susceptible to immunotherapy approaches and its test is suitable for diagnosis of GC tumor stage (Ratti et al., 2018). Through the MSI characteristic, we assessed the relationship with immune response and prognosis. The result of immune exclusion suggested that low-risk groups are more promising treatments for immunotherapy. The result of MSI indicated that there was a significant relationship between immune response and prognosis. Based on the above results, we confirmed that the HSPRGs-based prediction signature can be further developed as a reliable biomarker for the treatment of GC.

In addition to immunotherapy, chemotherapy is also crucial for improving the prognosis of GC, and is typically used in the treatment of advanced GC patients. However, most of the chemotherapy drugs for GC are cytotoxic and have serious adverse reactions, and patients have gradually developed resistance to chemotherapy drugs, which directly affect the efficacy of GC. In our study, we used GDSC to predict the sensitivity of subgroups of GC patients to different drugs. Our results show that Hippo-related signatures differ significantly among different drugs. Patients in the high-risk group were more sensitive to Bortezomib, Doxorubicin, Etoposide, Imatinib, Lapatinib, Paclitaxel, Rapamycin, and Sunitinib. Bortezomib, Doxorubicin, Lapatinib and Paclitaxel are currently the first-line chemotherapeutic drugs for the clinical treatment of GC (Jatoi et al., 2008). Imatinib and is particularly effective in the treatment of gastrointestinal stromal tumours and significantly improve the survival rate of patients (Blay et al., 2021). Lapatinib selectively inhibits HER2-amplified gastric cancer cells (Wainberg et al., 2010), and Rapamycin and Sunitinib target angiogenesis, significantly inhibiting tumor angiogenesis *in vivo* (Lang et al., 2007; Lyros et al., 2010). The chemotherapy drugs are generally used in combination to enhance the treatment effect and reduce the drug resistance of patients. For example, chidamide combined with bortezomib has the effect of anti-cancer GC (Zhang et al., 2020). However, the specific mechanism and efficacy of these drugs in GC still require further explored.

Overall, the prognostic signature we constructed is the first novel Hippo pathway-related prognostic model, which provides a strong rationale for the development of Hippo pathway-related biomarkers and therapeutic targets. In addition, contrasting with other models, we constructed a prognostic nomogram, adding immunotherapy and chemotherapeutic drug treatment responses, which had better performance in predicting patient clinical survival and response to immune and drug therapy. In our study, there are still limitations. The prognostic signature was constructed and validated based on the TCGA and GEO database, data in which are incomplete. Moreover, based on retrospective analysis, our research lacked prospective clinical trials and corresponding clinical experimental support.

## 5 Conclusion

In consequence, we successfully construct and validate a novel prognostic signature associated with HSPRGs in GC. Our findings suggest that the hippo-associated signature might facilitate clinical prognosis prediction and medication guidance in individualized management of GC.

## Data Availability

The datasets presented in this study can be found in online repositories. The names of the repository/repositories and accession number(s) can be found in the article/supplementary material.

## Abbreviations

GC, Gastric cancer; TCGA, The Cancer Genome Atlas; GEO, Gene Expression Omnibus; OS, Overall survival; HSPRGs, Hippo signaling pathway-Related Genes; KEGG, Kyoto Encyclopedia of Genes and Genomes; PPI, Protein-protein-interaction; LASSO, Least absolute shrinkage and selection operator; ROC, Receiver operating characteristic; GSEA, Gene set enrichment analysis; AUC, Area under the curve; TIDE, Tumor Immune Dysfunction and Exclusion; MSI, Microsatellite instability; PI3K/AKT, Phosphatidylinositol 3-kinase/protein kinase B; Wnt, Wingless-type MMTV integration site; DLG3, discs large MAGUK scaffold protein 3; TGFB3, transforming growth factor beta 3; TGFBR1, transforming growth factor beta receptor 1; FZD6, frizzled class receptor 6; CTNNB1, catenin beta 1; GSK3B, glycogen synthase kinase 3 beta, YAP, Yes-associated protein; TAZ, transcriptional co-activator with PDZ-binding motif.

## References

[B1] Blay J. Y. Kang Y. K. Nishida T. von Mehren M. (2021). Gastrointestinal stromal tumours. Nat. Rev. Dis. Prim. 7, 22. 10.1038/s41572-021-00254-5 33737510

[B2] Caruso S. Patriti A. Roviello F. De Franco L. Franceschini F. Coratti A. (2016). Laparoscopic and robot-assisted gastrectomy for gastric cancer: Current considerations. World J. Gastroenterol. 22, 5694–5717. 10.3748/wjg.v22.i25.5694 27433084 PMC4932206

[B3] Chen F. B. Wu P. Zhou R. Yang Q. X. Zhang X. Wang R. R. (2020). LINC01315 impairs microRNA-211-dependent DLG3 downregulation to inhibit the development of oral squamous cell carcinoma. Front. Oncol. 10, 556084. 10.3389/fonc.2020.556084 33117688 PMC7549330

[B4] Chen J. Miao L. Jin G. Ren C. Ke Q. Qian Y. (2014). TGFBR1 tagging SNPs and gastric cancer susceptibility: A two-stage case-control study in Chinese population. Mol. Carcinog. 53, 109–116. 10.1002/mc.21954 22911926

[B5] Chen Z. F. Wang J. Yu Y. Wei W. (2020). MicroRNA-936 promotes proliferation and invasion of gastric cancer cells by down-regulating FGF2 expression and activating P13K/Akt signaling pathway. Eur. Rev. Med. Pharmacol. Sci. 24, 6707–6715. 10.26355/eurrev_202006_21658 32633361

[B6] Geeleher P. Cox N. Huang R. S. (2014). pRRophetic: an R package for prediction of clinical chemotherapeutic response from tumor gene expression levels. PLoS One 9, e107468. 10.1371/journal.pone.0107468 25229481 PMC4167990

[B7] Grech G. Zhan X. Yoo B. C. Bubnov R. Hagan S. Danesi R. (2015). EPMA position paper in cancer: Current overview and future perspectives. EPMA J. 6, 9. 10.1186/s13167-015-0030-6 25908947 PMC4407842

[B8] Gu C. Chen J. Dang X. Chen C. Huang Z. Shen W. (2021). Hippo pathway core genes based prognostic signature and immune infiltration patterns in lung squamous cell carcinoma. Front. Oncol. 11, 680918. 10.3389/fonc.2021.680918 33996611 PMC8117235

[B9] Jahangir E. Polin N. (2016). Cardiac follow-up of cancer survivors. Eur. Heart J. 37, 2745–2747. 10.1093/eurheartj/ehw362 27694542

[B10] Jatoi A. Dakhil S. R. Foster N. R. Ma C. Rowland K. M. Jr. Moore D. F. Jr. (2008). Bortezomib, paclitaxel, and carboplatin as a first-line regimen for patients with metastatic esophageal, gastric, and gastroesophageal cancer: Phase II results from the north central cancer treatment group (N044B). J. Thorac. Oncol. 3, 516–520. 10.1097/JTO.0b013e31816de276 18449005 PMC3929582

[B11] Jiang P. Gu S. Pan D. Fu J. Sahu A. Hu X. (2018). Signatures of T cell dysfunction and exclusion predict cancer immunotherapy response. Nat. Med. 24, 1550–1558. 10.1038/s41591-018-0136-1 30127393 PMC6487502

[B12] Kang W. Cheng A. S. Yu J. To K. F. (2016). Emerging role of Hippo pathway in gastric and other gastrointestinal cancers. World J. Gastroenterol. 22, 1279–1288. 10.3748/wjg.v22.i3.1279 26811664 PMC4716037

[B13] Katoh M. (2005). WNT/PCP signaling pathway and human cancer (review). Oncol. Rep. 14, 1583–1588. 10.3892/or.14.6.1583 16273260

[B14] Kim S. H. Jin H. Meng R. Y. Kim D. Y. Liu Y. C. Chai O. H. (2019). Activating hippo pathway via rassf1 by ursolic acid suppresses the tumorigenesis of gastric cancer. Int. J. Mol. Sci. 20, 4709. 10.3390/ijms20194709 31547587 PMC6801984

[B15] Kriz V. Korinek V. (2018). Wnt, RSPO and hippo signalling in the intestine and intestinal stem cells. Genes (Basel) 9, 20. 10.3390/genes9010020 29316729 PMC5793173

[B16] Lang S. A. Gaumann A. Koehl G. E. Seidel U. Bataille F. Klein D. (2007). Mammalian target of rapamycin is activated in human gastric cancer and serves as a target for therapy in an experimental model. Int. J. Cancer 120, 1803–1810. 10.1002/ijc.22442 17230506

[B17] Laverty H. G. Wakefield L. M. Occleston N. L. O'Kane S. Ferguson M. W. (2009). TGF-beta3 and cancer: A review. Cytokine Growth Factor Rev. 20, 305–317. 10.1016/j.cytogfr.2009.07.002 19656717 PMC7294566

[B18] Li D. Hu X. Yu S. Deng S. Yan M. Sun F. (2020). Silence of lncRNA MIAT-mediated inhibition of DLG3 promoter methylation suppresses breast cancer progression via the Hippo signaling pathway. Cell Signal 73, 109697. 10.1016/j.cellsig.2020.109697 32593652

[B19] Li K. Zhang A. Li X. Zhang H. Zhao L. (2021). Advances in clinical immunotherapy for gastric cancer. Biochim. Biophys. Acta Rev. Cancer 1876, 188615. 10.1016/j.bbcan.2021.188615 34403771

[B20] Lichtman M. K. Otero-Vinas M. Falanga V. (2016). Transforming growth factor beta (TGF-beta) isoforms in wound healing and fibrosis. Wound Repair Regen. 24, 215–222. 10.1111/wrr.12398 26704519

[B21] Lin E. Kuo P. H. Liu Y. L. Yang A. C. Tsai S. J. (2017). Transforming growth factor-beta signaling pathway-associated genes SMAD2 and TGFBR2 are implicated in metabolic syndrome in a Taiwanese population. Sci. Rep. 7, 13589. 10.1038/s41598-017-14025-4 29051557 PMC5648797

[B22] Liu B. Xu Y. Zhang L. Yang X. Chen L. Liu Y. (2021). Hypermethylation of DLG3 promoter upregulates RAC1 and activates the PI3K/AKT signaling pathway to promote breast cancer progression. Evid. Based Complement. Altern. Med. 2021, 8428130. 10.1155/2021/8428130 PMC8577895 34765009

[B23] Liu J. Li P. Wang R. Li J. Zhang M. Song Z. (2019). High expression of DLG3 is associated with decreased survival from breast cancer. Clin. Exp. Pharmacol. Physiol. 46, 937–943. 10.1111/1440-1681.13132 31271664 PMC6771499

[B24] Liu Y. Wu J. Huang W. Weng S. Wang B. Chen Y. (2020). Development and validation of a hypoxia-immune-based microenvironment gene signature for risk stratification in gastric cancer. J. Transl. Med. 18, 201. 10.1186/s12967-020-02366-0 32410620 PMC7226948

[B25] Lyros O. Mueller A. Heidel F. Schimanski C. C. Gockel I. Galle P. R. (2010). Analysis of anti-proliferative and chemosensitizing effects of sunitinib on human esophagogastric cancer cells: Synergistic interaction with vandetanib via inhibition of multi-receptor tyrosine kinase pathways. Int. J. Cancer 127, 1197–1208. 10.1002/ijc.25137 20039326

[B26] Mohamed R. H. Abu-Shahba N. Mahmoud M. Abdelfattah A. M. H. Zakaria W. ElHefnawi M. (2019). Co-Regulatory network of oncosuppressor miRNAs and transcription factors for pathology of human hepatic cancer stem cells (HCSC). Sci. Rep. 9, 5564. 10.1038/s41598-019-41978-5 30944375 PMC6447552

[B27] Mootha V. K. Lindgren C. M. Eriksson K. F. Subramanian A. Sihag S. Lehar J. (2003). PGC-1alpha-responsive genes involved in oxidative phosphorylation are coordinately downregulated in human diabetes. Nat. Genet. 34, 267–273. 10.1038/ng1180 12808457

[B28] Moroishi T. Hayashi T. Pan W. W. Fujita Y. Holt M. V. Qin J. (2016). The hippo pathway kinases LATS1/2 suppress cancer immunity. Cell 167, 1525–1539. e1517. 10.1016/j.cell.2016.11.005 27912060 PMC5512418

[B29] Naef M. Ishiwata T. Friess H. Buchler M. W. Gold L. I. Korc M. (1997). Differential localization of transforming growth factor-beta isoforms in human gastric mucosa and overexpression in gastric carcinoma. Int. J. Cancer 71, 131–137. 10.1002/(sici)1097-0215(19970410)71:2<131::aid-ijc1>3.0.co;2-1 9139831

[B30] Qian X. He L. Hao M. Li Y. Li X. Liu Y. (2021). YAP mediates the interaction between the Hippo and PI3K/Akt pathways in mesangial cell proliferation in diabetic nephropathy. Acta Diabetol. 58, 47–62. 10.1007/s00592-020-01582-w 32816106

[B31] Qiao Y. Li T. Zheng S. Wang H. (2018). The Hippo pathway as a drug target in gastric cancer. Cancer Lett. 420, 14–25. 10.1016/j.canlet.2018.01.062 29408652

[B32] Ratti M. Lampis A. Hahne J. C. Passalacqua R. Valeri N. (2018). Microsatellite instability in gastric cancer: Molecular bases, clinical perspectives, and new treatment approaches. Cell Mol. Life Sci. 75, 4151–4162. 10.1007/s00018-018-2906-9 30173350 PMC6182336

[B33] Seeneevassen L. Dubus P. Gronnier C. Varon C. (2022). Hippo in gastric cancer: From signalling to therapy. Cancers (Basel) 14, 2282. 10.3390/cancers14092282 35565411 PMC9105983

[B34] Shao Y. Jia H. Li S. Huang L. Aikemu B. Yang G. (2021). Comprehensive analysis of ferroptosis-related markers for the clinical and biological value in gastric cancer. Oxid. Med. Cell Longev. 2021, 7007933. 10.1155/2021/7007933 34745421 PMC8566081

[B35] Smyth E. C. Nilsson M. Grabsch H. I. van Grieken N. C. Lordick F. (2020). Gastric cancer. Lancet 396, 635–648. 10.1016/S0140-6736(20)31288-5 32861308

[B36] Thrift A. P. El-Serag H. B. (2020). Burden of gastric cancer. Clin. Gastroenterol. Hepatol. 18, 534–542. 10.1016/j.cgh.2019.07.045 31362118 PMC8859863

[B37] Wainberg Z. A. Anghel A. Desai A. J. Ayala R. Luo T. Safran B. (2010). Lapatinib, a dual EGFR and HER2 kinase inhibitor, selectively inhibits HER2-amplified human gastric cancer cells and is synergistic with trastuzumab *in vitro* and *in vivo* . Clin. Cancer Res. 16, 1509–1519. 10.1158/1078-0432.CCR-09-1112 20179222

[B38] Wong M. C. S. Huang J. Chan P. S. F. Choi P. Lao X. Q. Chan S. M. (2021). Global incidence and mortality of gastric cancer, 1980-2018. JAMA Netw. Open 4, e2118457. 10.1001/jamanetworkopen.2021.18457 34309666 PMC8314143

[B39] Wu Y. Shen L. Liang X. Li S. Ma L. Zheng L. (2019). Helicobacter pylori-induced YAP1 nuclear translocation promotes gastric carcinogenesis by enhancing IL-1β expression. Cancer Med. 8, 3965–3980. 10.1002/cam4.2318 31145543 PMC6639191

[B40] Xu X. Lu Y. Wu Y. Wang M. Wang X. Wang H. (2021). A signature of seven immune-related genes predicts overall survival in male gastric cancer patients. Cancer Cell Int. 21, 117. 10.1186/s12935-021-01823-0 33602220 PMC7891008

[B41] Yan H. Qiu C. Sun W. Gu M. Xiao F. Zou J. (2018). Yap regulates gastric cancer survival and migration via SIRT1/Mfn2/mitophagy. Oncol. Rep. 39, 1671–1681. 10.3892/or.2018.6252 29436693 PMC5868403

[B42] Yan J. Liu T. Zhou X. Dang Y. Yin C. Zhang G. (2016). FZD6, targeted by miR-21, represses gastric cancer cell proliferation and migration via activating non-canonical wnt pathway. Am. J. Transl. Res. 8, 2354–2364. 27347343 PMC4891448

[B43] Yong J. Li Y. Lin S. Wang Z. Xu Y. (2021). Inhibitors targeting YAP in gastric cancer: Current status and future perspectives. Drug Des. Devel Ther. 15, 2445–2456. 10.2147/DDDT.S308377 PMC8203099 34140763

[B44] Yoon S. J. Park J. Shin Y. Choi Y. Park S. W. Kang S. G. (2020). Deconvolution of diffuse gastric cancer and the suppression of CD34 on the BALB/c nude mice model. BMC Cancer 20, 314. 10.1186/s12885-020-06814-4 32293340 PMC7160933

[B45] Zhang W. Niu J. Ma Y. Yang X. Cao H. Guo H. (2020). The synergistic antitumor activity of chidamide in combination with bortezomib on gastric cancer. Onco Targets Ther. 13, 3823–3837. 10.2147/OTT.S240721 32440150 PMC7213427

